# Factors associated with insomnia, anxiety, and depression among antenatal women in China: A cross-sectional hospital-based study

**DOI:** 10.1371/journal.pone.0344846

**Published:** 2026-03-24

**Authors:** Qiaoling Liao, Ruoxin Fan, Dandan Zheng, Zuowei Li, Xianmei Yang, Jun Liu, Yaozhi Hu

**Affiliations:** 1 The Second Department of Severe Psychiatry, The Third Hospital of Mianyang, Sichuan Mental Health Center, Mianyang, China; 2 Mental Health Prevention and Treatment Division, The Third Hospital of Mianyang, Sichuan Mental Health Center, Mianyang, China; 3 Faculty of Nursing, Mahidol University, Bangkok, Thailand; 4 Nursing Department, The Third Hospital of Mianyang, Sichuan Mental Health Center, Mianyang, China; 5 The First Department of Severe Psychiatry, The Third Hospital of Mianyang, Sichuan Mental Health Center, Mianyang, China; National Research Centre, EGYPT

## Abstract

**Background:**

Mental health challenges, including insomnia, anxiety, and depression, are common among antenatal women and can affect both maternal and fetal outcomes. This study explores the determinants of these conditions in antenatal women in China, aiming to inform the design of mental health interventions and preventive strategies for this population.

**Methods:**

A cross-sectional survey design was employed in this hospital-based study targeting antenatal women at a tertiary hospital in China, conducted from May 2024 to March 2025 during routine antenatal visits. Validated questionnaires assessed insomnia, anxiety, and depression. Multiple linear and logistic regression analyses identified factors associated with symptom severity and occurrence, while Structural Equation Modeling (SEM) was used to explore the relationships and mediating effects between biological and social factors, insomnia, anxiety, and depression.

**Results:**

The participants had a mean age of 31.48 ± 6.94 years, with most being married (90.7%), living in urban areas (74.3%), and having undergraduate/college education (45.8%). Significant predictors of insomnia included geographical location, with those in central (OR = 1.818, 95% CI: 1.500–2.204) and southern areas (OR = 1.368, 95% CI: 1.143–1.637) showing higher odds compared to the northern region. Living in rural areas (OR = 0.796, 95% CI: 0.718–0.845) and higher education levels (OR = 1.544, 95% CI: 1.012–2.355) were associated with lower odds. Other significant factors included the number of live births and household composition. For anxiety, older age (OR = 0.955, 95% CI: 0.937–0.973) and rural living (OR = 0.675, 95% CI: 0.539–0.845) decreased odds, while living with others (OR = 3.726, 95% CI: 2.463–5.639) increased the risk. Significant predictors of depression included geographical location (central areas: OR = 1.508, 95% CI: 1.106–2.055), income level, and number of live births. The logistic regression Area Under the Curve (AUC) were 0.579 for insomnia, 0.603 for anxiety, and 0.567 for depression. SEM demonstrated an excellent model fit (CFI = 0.994, TLI = 0.999, RMSEA = 0.014). Insomnia was strongly predicted by geographic location, education, and number of live births. In turn, insomnia significantly predicted anxiety (β = 0.741) and depression (β = 0.138). The model explained 54.9% of the variance in anxiety and 70.6% of the variance in depression, indicating partial mediation.

**Conclusion:**

This study identifies the multidimensional factors influencing antenatal women’s insomnia, anxiety, and depression in China, particularly highlighting the roles of geographical location, current living situation, and household composition. These factors were consistently associated with all three outcomes. Targeted interventions targeting these specific risk factors are recommended to improve the mental health of antenatal women.

## 1. Introduction

The antenatal period, defined as the timeframe from conception to birth [1], is a critical phase for both maternal and fetal outcomes [2]. During this period, physiological adaptations occur to support fetal development and prepare the maternal body for childbirth [3]. These physiological changes can significantly affect maternal psychological well-being [4], potentially leading to mental health challenges such as insomnia [5], anxiety [6], and depression [7]. Perinatal depression affects one in five women globally [8]. The severity of psychological distress during pregnancy can negatively impact maternal health and fetal development, which may led to complications such as low birth weight, preterm delivery, and behavioral problems later in life [9].

Epidemiological data highlight a concerning prevalence of sleep disturbances, depression, and anxiety within this population. The data show approximately 50% of antenatal women experience suboptimal sleep quality [10]. A comprehensive review of 523 longitudinal studies established an average antenatal depression prevalence of 17%, with approximately 7% of antenatal women continuing to experience symptoms postpartum in Ethiopia [11], often accompanied by other mental health comorbidities [12]. Gestational depression prevalence ranges from 5% to 30% in developed countries (mostly 10% to 15%) and around 20% in developing countries, typically higher than in developed nations [4]. Systematic reviews have revealed that the prevalence of self-reported anxiety symptoms ranges from 13.6% to 22.8% in the first trimester, whereas the prevalence of clinically diagnosed anxiety disorders is approximately 15.2% [13]. The prevalence of anxiety during the antenatal period among women ranges from 4.4% to 39% across different populations [14], with European studies reporting prevalence rates between 0.3% and 10.8% [15], with comorbid anxiety and depression ranging from approximately 3.6% to 40.2% [16]. Australian data indicate that 20.9% of antenatal women experience anxiety symptoms [17]. In China, the prevalence of insomnia reaches 59.8% among women in the third-trimester [18]. Recent studies show that antenatal mental disorders are more prevalent than postpartum conditions [19]. Early diagnosis and intervention can improve outcomes for both mother and child and may reduce the risk of postpartum depression [20]. These findings underscore the critical need for targeted research to address sleep disturbances, depression, and anxiety during pregnancy.

Studies have revealed that stress [21], increased maternal age [22], education level [23], lifestyle [24], optimism [25], sexual partner support [26], history of mental illness [27], social support [28], and the COVID-19 pandemic [29] are factors affecting maternal mental health. While numerous studies have identified key factors influencing maternal mental health, significant gaps persist in understanding their interactions, context-specific impacts, and implications for targeted interventions. Stressors [9] like financial difficulties or trauma are linked to higher antenatal and postpartum depression risk due to hormonal and emotional imbalances [30]. However, gaps exist in understanding how cumulative stress interacts with protective factors [31], especially in low-resource settings [32]. Advanced maternal age increases depression risk due to pregnancy complications and societal pressures [33], but its relationship with socioeconomic status remains unclear [34]. Maternal education and economic status are often overlooked [35], yet lower education attainment correlates with higher depression risk [36]. Research gaps include the intersection of education with cultural factors in low- and middle-income countries (LMICs) [37]. Unhealthy lifestyles exacerbate depression [38], but causal pathways and intervention efficacy remain underexplored [39–41]. Lower optimism increases depression risk through pessimistic biases [42], with few studies examining optimism in high-stress environments [43]. Unstable sexual partnerships raise depression risk due to relationship instability or violence [44], but this issue is underrepresented in research from LMICs [45]. Prior psychiatric conditions [46] predict depression recurrence [47], and gaps in early identification hinder prevention. Inadequate social support [48] increases depression risk [49]. The pandemic exacerbated depression due to isolation and healthcare disruption, with potentially lasting effects [50]. Despite extensive documentation, gaps remain in understanding the interactions among there factors, cultural variations, and effective intervention strategies.

The biopsychosocial model (BPSM), proposed by George L. Engel in 1977, proposes that health outcomes arise from the dynamic interplay of biological, psychological, and social factors [51]. In the context of antenatal women, the BPSM illustrates how pregnancy’s physiological shifts interact with psychological vulnerability (such as depression and anxiety) and social factors. This framework guided the selection of outcome indicators and influencing factors in this study. Specifically, the model helps explain how physiological changes during pregnancy interact with psychological vulnerabilities and social conditions. This study was conducted in Southwest China to examine the key factors associated with insomnia, depression, and anxiety among antenatal women. By identifying key risk factors such as socioeconomic status and prior mental health history, health care providers can implement targeted screening protocols, thereby enhancing early diagnosis and proactive management of antenatal mental health issues [52]. In addition, the findings are expected to benefit the local community by by identifying region-specific risk factors and providing a valuable reference for developing culturally sensitive and evidence-based interventions that can be integrated into routine antenatal care protocols. This study addresses the following research questions: (1) What are the prevalence and sociodemographic determinants of insomnia, anxiety, and depression among antenatal women in Southwest China? (2) How do these conditions interrelate within a biopsychosocial framework? The analyses were structured to directly address these research questions and inform targeted interventions.

## 2. Materials and methods

### 2.1 Study setting and design

This study employed a cross-sectional survey design. Data were collected from pregnant women receiving antenatal care at a tertiary hospital in a city in southwestern China between May 2024 and March 2025. A cluster sampling approach was employed to facilitate efficient data collection within the hospital setting and to minimize the temporal and financial costs associated with broad geographical distribution [53]. The hospital’s antenatal care clinics were divided into clusters based on daily clinic sessions, with clusters randomly selected proportion to their average patient volume. Within each selected cluster, all eligible women who met the predefined inclusion criteria during the study period were enrolled consecutively to ensure representativeness and reduce selection bias. Each questionnaire required approximately 30 minutes to complete. Participants completed the questionnaires independently, and a researcher was present to clarify any questions if necessary. The questionnaires were distributed and collected on site, allowing researchers to immediately review them for completeness and ensure data accuracy. 

### 2.2 Study participants

The inclusion criteria were as follows: (1) women aged ≥16 years and (2) those who provided written informed consent. The exclusion criteria were as follows: (1) patients with a diagnosis of severe psychiatric disorders (schizophrenia, schizoaffective disorder, delusional disorder, bipolar disorder, psychotic disorder due to epilepsy, or intellectual developmental disorder with co-occurring mental disorder) or severe cognitive impairment, as systematically assessed during hospital admission using standardized diagnostic criteria from the Diagnostic and Statistical Manual of Mental Disorders, Fifth Edition (DSM-5), ensuring that participants with depression alone were not excluded; and (2) patients who were illiterate or unable to perform basic reading tasks.

### 2.3 Study tools

#### 2.3.1 Demographic data.

The general information questionnaire was developed based on an extensive literature review and experts consultations. This approach facilitated better control of potential confounding variables in subsequent data analyses, thereby strengthening the scientific rigor and validity of the research findings [54]. Demographic information was collected using a self-administered questionnaire completed by participants during their antenatal care visit at the time of enrolment. The questionnaire included the following variables: age, marital status, geographical location, current living situation, educational attainment, residential area, income level, pregnancy status, number of live births, employment status, gestational age, expected mode of delivery and individuals the patient lives with. In the study, pregnancy status was defined as the total number of times a woman had been pregnant, including the current one. Number of live births was defined as the total number of previous pregnancies that resulted in a live birth. These variables were analyzed separately to distinguish between the physiological and psychological impacts of total pregnancy experiences versus successful live births.

#### 2.3.2 Insomnia.

The Insomnia Severity Index (ISI) is a self-report questionnaire developed by Charles M. Morin and Colin A. Espie in 1993 to assess insomnia severity [55]. The Chinese version was validated in 2013 [56]. The scale includes seven items on a 5-point Likert scale (0 = no symptoms to 4 = very severe) assessing perceived insomnia severity, sleep dissatisfaction, interference with daily functioning, and distress related to sleep problems, with scores ranging from 0–28 (0–7: no insomnia; 8–14: subthreshold; 15–21: moderate; 22–28: severe). The ISI has excellent psychometric properties, including high internal consistency (Cronbach’s α = 0.90–0.91 in the original validation [57]; α = 0.81 in the Chinese version [58]), test–retest reliability (r = 0.74–0.83) [59], and convergent validity with sleep diaries/polysomnography (r = 0.65–0.80) [52]. Participants completed the ISI as part of the anonymous self-administered questionnaire during routine antenatal clinic visits. Based on the cutoff points recommended by the scale developers and existing literature [60], an ISI total score ≥8 was used to define the presence of insomnia, whereas scores <8 indicated no clinically significant insomnia, consistent with the standard proposed by Charles M. Morin (2001), in which scores of 0–7 indicate insomnia without clinical significance.”

#### 2.3.3 Anxiety.

The Generalized Anxiety Disorder-7 (GAD-7) scale, developed by Robert L. Spitzer (2006) [61] and adapted into Chinese in 2010 [62], is a concise, self-report tool for screening [63] for generalized anxiety disorder [64]. It uses a 4-point Likert scale (0 = not at all to 3 = nearly every day) across seven items assessing symptoms such as nervousness, inability to control worrying, difficulty relaxing, irritability, and excessive worry. The total score ranges from 0 to 21, with cut-offs of 0−4 (minimal), 5−9 (mild), 10−14 (moderate), and 15−21 (severe). The Chinese version demonstrates strong reliability (Cronbach’s α = 0.84) [65] and validity, including good convergent validity with other anxiety measures (r > 0.70) and discriminant validity against depression scales [62]. Participants completed the GAD-7 as part of the anonymous self-administered questionnaire described above. Based on the scale developers’ recommendations and existing literature [66], a GAD-7 total score ≥10 was used to indicate the presence of anxiety, whereas scores <10 indicated no clinically significant anxiety.

#### 2.3.4 Depression.

The Patient Health Questionnaire-9 (PHQ-9), developed by Kurt Kroenke (1999) [67], is widely used in clinical, epidemiological, and screening contexts [68]. Its Chinese version was translated and validated in 2014 [69]. This 9-item self-report scale assesses depressive symptoms over the past two weeks using a 4-point Likert scale (0 = not at all to 3 = nearly every day), covering anhedonia, depressed mood, sleep disturbances, fatigue, appetite changes, guilt, concentration difficulties, psychomotor changes, and suicidal thoughts. The total score ranges from 0−27, with severity classified as follows: 0−4 (minimal), 5−9 (mild), 10−14 (moderate), 15−19 (moderately severe), and 20−27 (severe). The PHQ-9 has strong psychometric properties, including Cronbach’s α values of 0.81 [70] and 0.84 among pregnant women [71], with good content and construct validity (sensitivity of 88% and specificity of 88% for major depression) [67]. Participants completed the PHQ-9 as part of the anonymous self-administered questionnaire described above. Based on the recommended screening threshold [72], a total PHQ-9 score ≥10 was used to indicate the presence of depression, whereas scores <10 indicated no clinically significant depression.

### 2.4 Sample size

The sample size was estimated using the standard formula for cross-sectional studies, commonly applied in epidemiological research [73]. Using a 95% confidence level, a 5% margin of error, and an anticipated insomnia prevalence of 59.8%, the minimum sample size was estimated to be 368 participants. However, due to the considerable variation in the prevalence of our three outcomes (insomnia, anxiety, and depression) observed in previous studies, we intentionally recruited a much larger sample to ensure sufficient statistical power for analyzing each condition. This proactive approach, accounting for factors such as the cluster sampling design and potential non-response, resulted in a final sample of 8,999 participants, significantly enhancing the study’s statistical reliability and generalizability.

### 2.5 Data analysis

Data analysis was performed using IBM SPSS Statistics 27.0 [74], following the established data management and analysis procedures. Prior to analysis, missing data were handled by imputing the mode for categorical variables and the mean for continuous variables. Descriptive statistics for all study variables were calculated, with categorical variables presented as absolute frequencies and percentages, and continuous variables described using appropriate measures of central tendency and dispersion. Specifically, normally distributed data were presented as mean ± standard deviation, and non-normally distributed data as median (interquartile range). For inferential analysis, chi-square tests were used to compare categorical data, and independent-sample t-tests were used for continuous variables meeting parametric assumptions. Multicollinearity was assessed using the variance inflation factor (VIF) prior to regression analysis. Logistic regression modes were used to identify the determinants of insomnia, depression, and anxiety in the pregnant women. Odds ratios (ORs) and 95% confidence intervals (CIs) were calculated. All analyses were performed in RStudio software, with a predefined significance threshold of p < 0.05 (two-tailed). The dataset was split into training and testing sets, with model performance assessed by evaluating predictions on the test set and calculating the area under the receiver operating characteristic curve (AUC) value. Additionally, Structural Equation Modeling (SEM) was used to examine the relationships between biological, psychological, and social factors influencing insomnia, anxiety, and depression.

#### 2.5.1 Conceptual framework for structural equation modeling.

To guide the SEM analysis, we developed a hypothesized conceptual framework based on the biopsychosocial model (BPSM) outlined in the Introduction. This model posits that sociodemographic factors (e.g., geographical location, income level, and living arrangements) act as upstream predictors influencing insomnia as a biopsychological entry point, which in turn mediates pathways to anxiety and depression. Specifically, we hypothesized that: (1) regional and sociodemographic variables directly predict insomnia due to their impact on stress and access to resources; (2) insomnia positively predicts anxiety (via physiological dysregulation); and (3) anxiety mediates the effect of insomnia on depression, with potential direct effects also present. This framework is supported by prior literature on perinatal mental health pathways. SEM was employed to test these directional relationships, while adjusting for covariates, in alignment with our research objective to elucidate the interdependencies among mental health outcomes.

### 2.6 Ethical considerations

The study protocol received ethical approval under number 202414 from the ethics committee of the tertiary hospital in Mianyang, China. Informed consent was obtained from participants (or from their legal guardians for those aged < 18 years) before data collection. Additional information regarding the ethical, cultural, and scientific considerations specific to inclusivity in global research is provided in the Supporting Information.

## 3. Results

### 3.1 Characteristics of the study population

Among the 9,100 questionnaires distributed, 8,999 valid responses were collected (response rate: 98.9%) (Table 1). The average age was 31.48 ± 6.94 years. Most women were married (8,166, 91%), lived in urban areas (6,685, 74%). The majority of participants had 0–2 previous live births (8,605, 94%). More than one third participants were on maternity leave (3,952, 44%), while 3,386 (38%) were employed. About half of the women lived with their husbands (4,123, 46%), while approximately one quarter lived with their parents (2,226, 25%) or parents-in-law (2,235, 25%).

**Table 1:** Characteristics of the participants (*n* = 8999).

Characteristics		n	%	P-value		
Age	Mean±SD	31.48 ± 6.94		Insomnia	Depression	Anxiety
				.800^*^	.020^*^	.143^*^
Marital status	Unmarried	373	4	<.001^#^	<.001^#^	.003^#^
	Married	8166	91			
	Divorced or other	460	5			
Geographical location	North area	857	10	<.001^#^	.101^#^	.016^#^
	Central area	3804	42			
	South area	4338	48			
Current living situation	Urban streets	4824	54	<.001^#^	.030^#^	.006^#^
	Townships and villages	4175	46			
Educational attainment	Illiterate	141	2	.018^#^	.144^#^	.021^#^
	Primary school	246	3			
	Junior high school	2287	25			
	Senior high school/ technical school	2095	23			
	Undergraduate/college	4121	46			
	Postgraduate/PhD	109	1			
Residential area (Hukou)	Urban	6685	74	.131^#^	.519^#^	.950^#^
	Rural	2314	26			
Income level	3000	3239	36	.609^#^	<.001^#^	.180^#^
	3000 - 5000	3381	38			
	≥ 5000	2379	26			
Pregnancy status	0	316	4	.035^#^	.427^#^	.576^#^
	1	3509	39			
	2	2880	32			
	3	1240	14			
	4	586	6			
	≥ 5	468	5			
Number of live births	0	1575	17	.066^#^	.938^#^	.003^#^
	1	4537	50			
	2	2493	27			
	3	331	4			
	4	34	1			
	≥ 5	29	1			
Employment status	Unemployed	1661	18	.004^#^	.210^#^	.409^#^
	Employed	3386	38			
	On maternity leave	3952	44			
Gestational age	1-12 weeks	3380	38	.522^#^	.175^#^	.806^#^
	13-27 weeks	2257	25			
	≥ 28 weeks	3362	37			
Expected mode of delivery	Both	5035	56	.005^#^	.033^#^	.056^#^
	Normal delivery	1636	18			
	Caesarean section	2328	26			
Who do you live with?	Husband	4123	46	<.001^#^	<.001^#^	<.001^#^
	Parents-in-law	2235	25			
	Parents	2226	25			
	Other	315	4			
Insomnia	No	6441	72			
	Yes	2558	28			
Anxiety	No	8585	95			
	Yes	414	5			
Depression	No	8239	92			
	Yes	760	8			

*Abbreviation:* SD, Standard Deviation.

***Note:*** ^#^ Indicates the use of the Chi-square test; ^*^ indicates the use of the Independent Samples T-Test.

### 3.2 Bivariate analysis of insomnia, anxiety and depression

The associations of each covariate with insomnia are shown in Table 2. Our analysis revealed that being married was associated with 27.2% lower odds of insomnia (odds ratio, OR 0.728 [95% confidence interval, CI: 0.585–0.906]). Compared with individuals living in northern areas, those living in central areas had 95.2% higher odds of insomnia (OR 1.952 [95% CI: 1.625–2.346]), whereas those living in southern areas had 43.4% higher odds (OR 1.434 [95% CI: 1.202–1.710]). Compared with urban residents, township and village residents had 22.9% lower odds of insomnia (OR 0.771 [95% CI: 0.703–0.846]). Individuals with a bachelor’s or associate degree had significantly greater odds of insomnia compared with illiterate individuals (OR 1.655 [95% CI: 1.094–2.503]). The number of pregnancies was associated with reduced odds of insomnia: one pregnancy lowered the odds by 31.1% (OR 0.689 [95% CI: 0.541–0.876]), two pregnancies by 33.1% (OR 0.669 [95% CI: 0.524–0.853]), and five or more pregnancies by 27.4% (OR 0.726 [95% CI: 0.535–0.984]). In contrast, the number of deliveries was associated with increased odds: one delivery was associated with 14.2% higher odds (OR 1.142 [95% CI: 1.004–1.299]), whereas five or more deliveries increased the odds by 157.5% (OR 2.575 [95% CI: 1.232–5.380]). Employment reduced the odds of insomnia by 16.1% (OR 0.839 [95% CI: 0.738–0.953]), and maternity leave further lowered the odds by 18.4% (OR 0.816 [95% CI: 0.720–0.924]). With respect to living arrangements, individuals living with their parents had 16.9% higher odds of insomnia compared with those living with their husbands (OR 1.169 [95% CI: 1.043–1.310]), whereas those living with others had 99.2% higher odds (OR 1.992 [95% CI: 1.576–2.518]). No significant associations were observed for the other variables.

**Table 2:** Analysis of insomnia.

		Bivariate analysis					Multivariate analysis				
Characteristics		B	P value	OR	95% CI		B	P value	OR	95% CI	
					Lower	Upper				Lower	Upper
Age		.002	.505	1.002	.996	1.009	−0.002	0.595	0.998	0.990	1.006
Marital status		<.001					0.029				
	−.318	.004	.728	.585	.906	−0.144	0.278	0.866	0.667	1.123	0.919
	.009	.952	1.009	.757	1.344	0.118	0.475	1.126	0.813	1.558	1.706
Geographical location		<.001					<0.001				
	.669	<.001	1.952	1.625	2.346	0.598	<0.001	1.818	1.500	2.204	2.302
	.360	<.001	1.434	1.202	1.710	0.313	<0.001	1.368	1.143	1.637	1.750
Current living situation											
	−.206	<.001	.771	.703	.846	−2.228	<0.001	0.796	0.718	0.845	0.925
Educational attainment		.019					0.194				
	.220	.390	1.246	.755	2.057	0.205	0.432	1.228	0.736	2.047	1.699
	.397	.063	1.488	.979	2.261	0.391	0.070	1.478	0.916	2.260	1.720
	.362	.091	1.437	.944	2.185	0.338	0.120	1.402	0.822	2.147	1.609
	.504	.017	1.655	1.094	2.503	0.434	0.044	1.544	1.012	2.355	1.155
	.366	.263	1.400	.777	2.524	0.279	0.364	1.321	0.915	2.048	1.865
Residential area(Hukou)											
	−.080	.131	.923	.832	1.024	0.098	0.103	1.103	0.980	1.241	1.244
Income level		.609					0.246				
	−.051	.348	.950	.854	1.057	−0.061	0.276	0.941	0.843	1.050	1.013
	−.044	.067	.957	.852	1.076	−0.104	0.101	0.901	0.796	1.020	1.146
Pregnancy status		.037					0.557				
	−.373	.002	.689	.541	.876	−0.190	0.212	0.827	0.614	1.114	1.566
	−403	.001	.669	.524	.853	−0.217	0.176	0.805	0.588	1.102	1.451
	−.314	.018	.731	.563	.948	−0.126	0.458	0.882	0.633	1.229	1.403
	−.282	.057	.754	.565	1.008	−0.091	0.618	0.913	0.638	1.306	2.127
	−.320	.039	.726	.535	.984	−0.187	0.330	0.830	0.569	1.208	1.940
Number of live births		.075					<0.001				
	.133	.044	1.142	1.004	1.299	0.258	0.001	1.294	1.106	1.514	1.513
	.060	.405	1.062	.922	.1.224	0.241	0.020	1.272	1.039	1.558	1.441
	.105	.435	1.111	.854	1.445	0.251	0.114	1.285	1.041	1.757	2.502
	−.007	.986	.993	.460	2.145	0.161	0.691	1.175	0.530	2.606	12.442
	.946	.012	2.575	1.232	5.380	1.196	0.002	3.305	1.527	7.155	9.085
Employment status		.005					0.599				
	−.175	.007	.839	.738	.953	−0.061	0.392	0.941	0.819	1.081	1.170
	−.204	.001	.816	.720	.924	−0.362	0.560	0.696	0.206	2.349	1.338
Gestational age		.522					0.866				
	.010	.866	1.010	.897	1.137	0.005	0.935	1.005	0.891	1.134	1.235
	.058	.279	1.060	.954	1.178	0.028	0.611	1.028	0.923	1.145	1.304
Excepted mode of delivery		.005					0.041				
	−.206	.201	.814	.717	.924	0.047	0.940	1.048	0.310	3.544	1.190
	−.006	.920	.994	.893	1.108	0.232	0.708	1.261	0.374	4.248	1.565
Who do you live with?		<.001					<0.001				
	.101	.079	1.107	.988	1.239	0.148	0.013	1.16	1.032	1.303	1.679
	.156	.007	1.169	1.043	1.310	0.158	0.008	1.172	1.042	1.317	1.529
	.689	<.001	1.992	1.576	2.518	0.585	<0.001	1.795	1.399	2.304	4.842

*Abbreviation:* OR: odds ratio; CI, confidence interval.

The associations of each covariate with anxiety are shown in Table 3. Our analysis revealed that with each additional year of age, the odds of anxiety decreased by 3% (OR 0.97 [95% CI: 0.954–0.986]). Compared with their unmarried counterparts, married individuals had 39.2% lower odds of anxiety (OR 0.608 [95% CI: 0.403–0.919]). Compared with individuals in the northern region, those in the central area had 55.2% higher odds of anxiety (OR 1.552 [95% CI: 1.047–2.302]). Additionally, residence in townships or villages was associated with a 24.4% lower odds of anxiety compared with urban residence (OR 0.756, 95% CI: 0.618–0.925).

**Table 3:** Analysis of anxiety.

		Bivariate analysis					Multivariate analysis				
Characteristics		B	P value	OR	95% CI		B	P value	OR	95% CI	
					Lower	Upper				Lower	Upper
Age		−0.031	<0.001	0.970	0.954	0.986	−0.046	<0.001	0.955	0.937	0.973
Marital status		0.003					0.146				
	−0.497	0.018	0.608	0.403	0.919	−0.229	0.373	0.795	0.480	1.317	0.919
	−0.002	0.994	0.998	0.584	1.706	0.136	0.670	1.146	0.613	2.142	1.706
Geographical location		0.017					0.012				
	0.44	0.029	1.552	1.047	2.302	0.467	0.027	1.596	1.055	2.414	2.302
	0.177	0.363	1.194	0.815	1.750	0.156	0.615	1.433	0.792	1.725	1.750
Current living situation											
	−0.28	0.006	0.756	0.618	0.925	−0.393	<0.001	0.675	0.539	0.845	0.925
Educational attainment		0.022					0.002				
	−0.376	0.416	0.687	0.277	1.699	−0.145	0.759	0.759	0.341	2.193	1.699
	−0.156	0.661	0.855	0.425	1.720	−0.120	0.740	0.887	0.436	1.805	1.720
	−0.227	0.527	0.797	0.395	1.609	−0.280	0.442	0.756	0.370	1.544	1.609
	−0.55	0.120	0.577	0.288	1.155	−0.694	0.056	0.506	0.245	1.018	1.155
	−0.582	0.344	0.559	0.167	1.865	−0.754	0.231	0.474	0.137	1.616	1.865
Residential area											
	−0.007	0.950	0.993	0.793	1.244	−0.061	0.640	0.941	0.731	1.213	1.244
Income level		0.181					0.433				
	−0.217	0.065	0.805	0.639	1.013	−0.152	0.207	0.859	0.678	1.088	1.013
	−0.111	0.379	0.895	0.698	1.146	−0.041	0.756	0.959	0.739	1.245	1.146
Pregnancy status		0.579					0.642				
	−0.078	0.772	0.772	0.546	1.566	0.391	0.257	1.478	0.752	2.903	1.566
	−0.162	0.553	0.851	0.499	1.451	0.316	0.383	1.372	0.674	2.796	1.451
	−0.238	0.418	0.788	0.442	1.403	0.160	0.680	1.173	0.550	2.501	1.403
	0.144	0.644	1.155	0.627	2.127	0.468	0.248	1.597	0.722	3.528	2.127
	0.013	0.968	1.014	0.529	1.940	0.267	0.530	1.306	0.568	3.008	1.940
Number of live births		0.070					0.021				
	0.13	0.370	1.370	0.857	1.513	0.173	0.327	1.189	0.841	1.682	1.513
	0.051	0.752	1.052	0.768	1.441	0.198	0.383	1.219	0.914	1.524	1.441
	0.401	0.127	1.494	0.892	2.502	0.576	0.076	1.778	0.942	3.356	2.502
	1.605	<0.001	4.978	1.992	12.442	1.653	0.001	5.225	1.990	14.299	12.442
	0.986	0.113	2.680	0.791	9.085	1.039	0.121	2.825	1.260	10.499	9.085
Employment status		0.410					0.724				
	−0.124	0.752	0.883	0.667	1.170	−0.125	0.422	0.883	0.652	1.196	1.170
	0.022	0.027	1.023	0.782	1.338	0.150	0.453	1.162	0.785	1.720	1.338
Gestational age		0.807					0.905				
	−0.047	0.724	0.955	0.737	1.235	−0.048	0.720	0.953	0.733	1.239	1.235
	0.039	0.736	1.040	0.829	1.304	0.009	0.940	1.009	0.801	1.270	1.304
Excepted mode of delivery		0.057					0.074				
	−1.109	0.450	0.897	0.676	1.190	−1.150	0.453	0.861	0.582	1.274	1.190
	0.224	0.050	1.251	1.000	1.565	−1.120	0.505	0.887	0.623	1.321	1.565
Who do you live with?		<0.001					<0.001				
	0.274	0.028	1.315	1.030	1.679	0.253	0.047	1.288	1.003	1.655	1.679
	0.17	0.192	1.192	0.918	1.529	0.225	0.090	1.252	0.966	1.623	1.529
	1.199	<0.001	3.318	2.274	4.842	1.315	<0.001	3.726	2.463	5.639	4.842

*Abbreviation:* OR: odds ratio; CI, confidence interval.

The associations of each covariate with depression are shown in Table 4. The results revealed that married women had significantly lower odds of depression, with a reduction of approximately 42.6% (OR 0.574 [95% CI: 0.420–0.783]). Compared with women living in northern China, central China had 47% higher odds of depreddon (OR 1.47 [95% CI: 1.095–1.974]). Antenatal women currently living in rural areas had significantly lower odds of depression, with a reduction of approximately 15.3% (OR 0.847 [95% CI: 0.729–0.984]). An income level of 3000–5000 RMB significantly reduced the odds of depression by approximately 28.6% (OR 0.714 [95% CI: 0.601–0.848]). An income level greater than 5000 RMB significantly reduced the odds of depression by approximately 26.2% (OR 0.738 [95% CI: 0.610–0.892]). Having four previous deliveries significantly increased the odds of depression by approximately 291.5% (OR 3.915 [95% CI: 1.745–8.773]). Expecting natural childbirth significantly reduced the odds of depression by approximately 24.8% (OR 0.752 [95% CI: 0.607–0.933]). Living with other family members significantly increased the odds of depression by approximately 20.8% (OR 1.208 [95% CI: 1.104–1.454]). Living alone significantly increased the odds of depression by approximately 222.6% (OR 3.226 [95% CI: 2.403–4.333]).

**Table 4:** Analysis of depression.

		Bivariate analysis					Multivariate analysis				
Characteristics		B	P value	OR	95% CI		B	P value	OR	95% CI	
					Lower	Upper				Lower	Upper
Age		−0.003	0.603	0.997	0.986	1.008	−0.012	0.066	0.988	0.976	1.001
Marital status		<0.001					0.020				
	−0.556	<0.001	0.574	0.420	0.783	−0.289	0.138	0.749	0.511	1.097	0.919
	−0.028	0.894	0.973	0.648	1.460	0.087	0.719	1.091	0.680	1.750	1.706
Geographical location		0.011					0.005				
	0.385	0.010	1.470	1.095	1.974	0.411	0.009	1.508	1.106	2.055	2.302
	0.193	0.183	1.213	0.913	1.611	0.159	0.281	1.281	0.878	1.565	1.750
Current living situation											
	−0.166	0.030	0.847	0.729	0.984	−0.245	0.004	0.783	0.662	0.926	0.925
Education attainment		0.147					0.096				
	0.675	0.107	1.965	0.864	4.466	0.770	0.070	2.159	0.938	4.970	1.699
	0.54	0.145	1.716	0.829	3.551	0.572	0.127	1.771	0.850	3.692	1.720
	0.464	0.212	1.591	0.767	3.300	0.474	0.207	1.607	0.769	3.358	1.609
	0.343	0.352	1.409	0.684	2.902	0.325	0.384	1.385	0.665	2.881	1.155
	0.132	0.801	1.805	0.401	3.250	0.145	0.790	1.156	0.399	3.349	1.865
Residential area											
	0.057	0.520	1.058	0.891	1.257	0.072	0.466	1.075	0.885	1.305	1.244
Income level		<0.001					<.001				
	−0.337	<0.001	0.714	0.601	0.848	−0.306	<.001	0.736	0.617	0.879	1.013
	−0.304	0.002	0.738	0.610	0.892	−0.291	0.004	0.747	0.612	0.913	1.146
Pregnancy status		0.429					0.544				
	−0.333	0.082	0.716	0.492	1.044	0.274	0.267	1.315	0.811	2.132	1.566
	−0.264	0.172	1.172	0.525	1.122	0.366	0.159	1.442	0.867	2.399	1.451
	−0.224	0.280	0.799	0.532	1.200	0.392	0.152	1.480	0.865	2.533	1.403
	−0.112	0.621	0.894	0.571	1.400	0.490	0.096	1.632	0.917	2.905	2.127
	−0.34	0.174	1.174	0.437	1.161	0.217	0.491	1.242	0.670	2.301	1.940
Number of live births		0.939					0.992				
	−0.08	0.443	0.924	0.754	1.132	−0.028	0.828	0.973	0.759	1.246	1.513
	−0.064	0.285	0.952	0.753	1.202	−0.055	0.737	0.947	0.688	1.303	1.441
	0.214	0.076	1.239	0.668	1.846	−0.084	0.740	0.919	0.559	1.512	2.502
	−0.312	0.001	3.915	1.745	8.773	0.271	0.630	1.311	0.435	3.952	12.442
	−0.168	0.121	1.785	0.354	3.681	0.038	0.953	1.039	0.293	3.681	9.085
Employment status		0.210					0.963				
	−0.061	0.558	0.941	0.767	1.154	0.031	0.784	1.032	0.825	1.291	1.170
	−0.169	0.103	0.845	0.690	1.034	0.018	0.882	1.010	0.801	1.274	1.338
Gestational age		0.715					0.773				
	0.066	0.503	1.068	0.881	1.293	0.065	0.515	1.067	.878	1.297	1.235
	0.064	0.468	1.066	0.897	1.266	0.051	0.570	1.052	.883	1.253	1.304
Excepted mode of delivery		0.034					0.109				
	−0.284	0.010	0.752	0.607	0.933	−1.286	0.301	0.818	0.581	1.164	1.190
	−0.038	0.666	0.962	0.808	1.146	−1.158	0.260	0.860	0.622	1.201	1.565
Who do you live with?		<0.001					<.001				
	0.017	0.864	1.017	0.840	1.230	0.033	0.740	1.034	.851	1.256	1.679
	0.189	0.045	1.208	1.104	1.454	0.211	0.028	1.235	1.023	1.490	1.529
	1.171	<0.001	3.226	2.403	4.333	1.136	<.001	3.114	2.260	4.290	4.842

*Abbreviation:* OR: odds ratio; CI, confidence interval.

### 3.3 Multivariate analysis of insomnia, anxiety and depression

The results indicated that, compared with individuals in the northern area, those in the central area had 81.8% higher odds of insomnia (OR 1.818 [95% CI: 1.500–2.204]). Similarly, those living in the southern area had 36.8% higher odds of insomnia (OR 1.368 [95% CI: 1.143–1.637]). Compared with those living in urban areas, individuals in townships and villages had 20.4% lower odds of insomnia (OR 0.796 [95% CI: 0.718–0.845]). Compared with illiterate individuals, individuals with an undergraduate or college had 54.4% higher odds of insomnia (OR 1.544 [95% CI: 1.012–2.355]). One previous birth significantly increased the odds of insomnia by 29.4% (OR 1.294 [95% CI: 1.106–1.514]). Two previous births increased the odds by 27.2% (OR 1.272 [95% CI: 1.039–1.558]). Five or more previous births were associated with 230.5% higher odds of insomnia (OR 3.305 [95% CI: 1.527–7.155]). Living with parents-in-law increased the odds of having insomnia by 16.0% (OR 1.160 [95% CI: 1.032–1.303]). Living with one's parents increased the odds by 17.2% (OR 1.172 [95% CI: 1.042–1.317]). Living with others significantly increased the odds by 79.5% (OR 1.795 [95% CI: 1.399–2.304]). (Table 2)

The results for anxiety indicated that individuals in the central area had 59.6% higher odds of anxiety (OR 1.596 [95% CI: 1.055–2.414]). Compared with those living in urban areas, individuals in townships and villages had 32.5% lower odds of anxiety (OR 0.675 [95% CI: 0.539–0.845]). Five or more previous births significantly increased the odds of anxiety by 422.5% (OR 5.225 [95% CI: 1.990–14.299]). Older age was associated with a reduced odds of anxiety (OR 0.955 [95% CI: 0.937–0.973]), indicating a 4.5% decrease in odds per additional year of age. (Table 3)

The results for depression indicated that, compared with those in northern China, individuals in central China had 50.8% higher odds of depression (OR 1.508 [95% CI: 1.106–2.055]). Compared with those living in urban areas, individuals in townships and villages had 21.7% lower odds of depression (OR 0.783 [95% CI: 0.662–0.926]). Individuals with an income of 3000–5000 RMB had 26.4% lower odds of depression (OR 0.736 [95% CI: 0.617–0.879]). An income of ≥5000 RMB was associated with 25.3% lower odds of depression (OR 0.747 [95% CI: 0.612–0.913]). Five or more previous births significantly increased the odds of depression by 211.4% (OR 3.114 [95% CI: 2.260–4.290]). Two previous births had 23.5% higher odds (OR 1.235 [95% CI: 1.023–1.490]). Living with other individuals significantly increased the odds of depression by 211.4% (OR 3.114 [95% CI: 2.260–4.290]). (Table 4)

### 3.4 Model explanatory power: Nagelkerke R² and Cox & Snell R² values

The results show that the model’s explanatory power is relatively weak for all three variables. Specifically, the Nagelkerke R² and Cox & Snell R² for depression (0.030 and 0.013, respectively), anxiety (0.044 and 0.014, respectively), and insomnia (0.027 and 0.019, respectively) indicate that the model explains only a small porportion of the variance in these outcomes. (Table 5)

**Table 5:** Nagelkerke and Cox & Snell Pseudo R² values.

Variable	Nagelkerke R²	Cox & Snell R²
**Depression**	0.030	0.013
**Anxiety**	0.044	0.014
**Insomnia**	0.027	0.019

### 3.5 Interrelationships between insomnia, anxiety, and depression

These results indicate significant positive associations between insomnia, depression, and anxiety, with varying strengths of correlation. Notably, the correlation between depression and anxiety was the strongest (Phi = 0.500). All associations were statistically significant (P < 0.05), highlighting the interrelatedness of these variables. (Table 6).

**Table 6:** Phi Coefficients and P-values for pairwise associations.

Variable	Phi	P-value
Insomnia * Depression	0.348	<.001
Insomnia * Anxiety	0.303	<.001
Depression * Anxiety	0.500	0.000

### 3.6 SEM result

**Fig 1:**
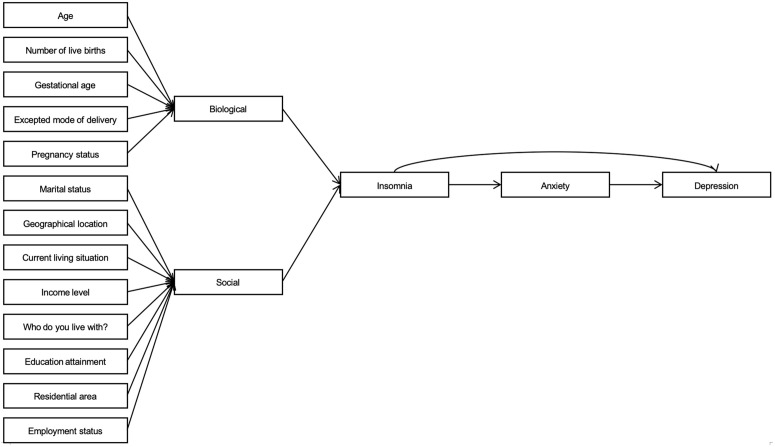
Chain mediation model based on the biopsychosocial framework.

The structural equation model demonstrated excellent fit to the data (CFI = 0.994 [robust: 0.991], TLI = 0.999, RMSEA = 0.014 [robust: 0.016, 90% CI: 0.010–0.018], SRMR = 0.002), despite a significant chi-square statistic (p < 0.001), which is common in large samples (N = 8999). Insomnia was significantly predicted by several covariates, including negative associations with age (β = −0.033, p = 0.037), current living situation (β = −0.098, p < 0.001), income level (β = −0.040, p = 0.008), and employment status (β = −0.093, p < 0.001), as well as positive associations with number of live births (marginally significant, β = 0.036, p = 0.053), expected mode of delivery (β = 0.063, p = 0.013), and living arrangements (“who do you live with?”) (β = 0.094, p < 0.001); other covariates (gestational age, pregnancy status, marital status, geographical location, education attainment, and residential area) showed no significant effects. The model explained a small proportion of the variance in insomnia (R² = 0.021). Anxiety was strongly and positively predicted by insomnia (β = 0.741, p < 0.001; R² = 0.549), while depression was positively predicted by both anxiety (β = 0.732, p < 0.001) and insomnia (β = 0.138, p < 0.001; R² = 0.706), suggesting partial mediation, whereby insomnia indirectly influences depression through anxiety. (Table 7 and Fig 1)

**Table 7:** Parameter estimates.

(Path/Parameter)	Estimate	Std.Err	Z-value	P-value	Std.all	Sig.
**Regressions on Insomnia**						
Age	−0.005	0.002	−2.090	0.037	−0.033	*
Number of live births	0.045	0.023	1.937	0.053	0.036	.
Gestational age	0.020	0.017	1.162	0.245	0.017	NS
Excepted mode of delivery	0.075	0.030	2.475	0.013	0.063	*
Pregnancy status	0.003	0.015	0.206	0.837	0.004	NS
Marital status	0.046	0.047	0.971	0.331	0.014	NS
Geographical location	−0.010	0.024	−0.414	0.679	−0.006	NS
Current living situation	−0.199	0.032	−6.191	0.000	−0.098	***
Income level	−0.052	0.019	−2.653	0.008	−0.040	**
Who do you live with?	0.104	0.016	6.500	0.000	0.094	***
Education attainment	−0.005	0.017	−0.320	0.749	−0.005	NS
Residential area	0.023	0.037	0.625	0.532	0.010	NS
Employment status	−0.126	0.034	−3.718	0.000	−0.093	***
**Regressions on Anxiety**						
Insomnia → Anxiety	0.737	0.018	40.603	0.000	0.741	***
**Regressions on Depression**						
Anxiety → Depression	0.732	0.046	15.870	0.000	0.732	***
Insomnia → Depression	0.138	0.039	3.556	0.000	0.138	***
**Thresholds**						
Insomnia	0.550	0.183	2.998	0.003	0.544	**
Anxiety	0.498	0.299	1.667	0.095	0.495	.
Depression	0.979	0.241	4.069	0.000	0.974	***
**Variances**						
Insomnia	1.000	–	–	–	0.979	–
Anxiety	0.456	–	–	–	0.451	–
Depression	0.297	–	–	–	0.294	–

*Note: ns = not significant;. = p < 0.10; = p < 0.05; = p < 0.01; = p < 0.001. Std.all is the standardized coefficient. R² values: Insomnia = 0.021, Anxiety = 0.549, Depression = 0.706.*

Structural equation model showing pathways from covariates through insomnia and anxiety to depression (N = 8999). Standardized path coefficients are shown; solid lines indicate significant paths (p < 0.05), dashed lines indicate non-significant or marginally significant paths. Model fit indices: CFI = 0.994, TLI = 0.999, RMSEA = 0.014, SRMR = 0.002.

### 3.7 Constructing a prediction model based on logistic regression results

The logistic regression model was used to identify significant predictors, defined as variables with p < 0.05 (the alpha level was set at 0.05 for statistical significance).

**Fig 2:**
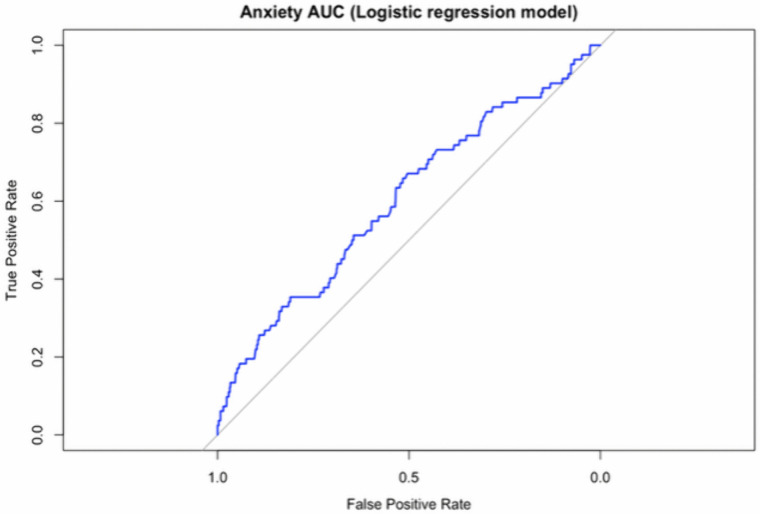
Receiver operating characteristic (ROC) curve for the logistic regression model predicting insomnia.

For insomnia, geographical location (overall), current living situation (townships and villages), number of live births (1, 2, and ≥ 5 births), education attainment (junior high school and undergraduate/college), expected mode of delivery (both modes), and living arrangement (“Who do you live with?”) (overall) were the most significant predictors. These factors were included in the logistic regression model (AUC = 0.579). (Fig 2)

The model included the following significant predictors (p < 0.05): geographical location (overall), current living situation (townships and villages), number of live births (1, 2, and ≥5), education attainment (junior high school and undergraduate/college), expected mode of delivery (both modes), and living arrangement (overall). The area under the curve (AUC) = 0.579.

**Fig 3:**
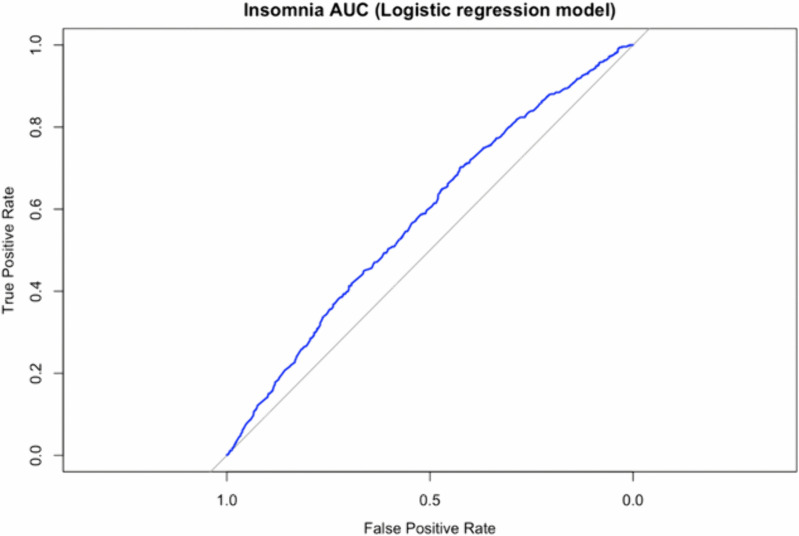
Receiver operating characteristic (ROC) curve for the logistic regression model predicting anxiety.

For anxiety, the same criterion (p < 0.05 from logistic regression) was applied to select age, geographical location (north and central area), current living situation (townships and villages), number of live births (0 and 4 births), and living arrangement (“Who do you live with?”) (overall) as significant predictors. These factors were included in the logistic regression model (AUC = 0.603). (Fig 3)

The model included the following significant predictors (p < 0.05): age, geographical location (north and central areas), current living situation (townships and villages), number of live births (0 and 4), and living arrangement (overall). The area under the curve (AUC) = 0.603.

**Fig 4:**
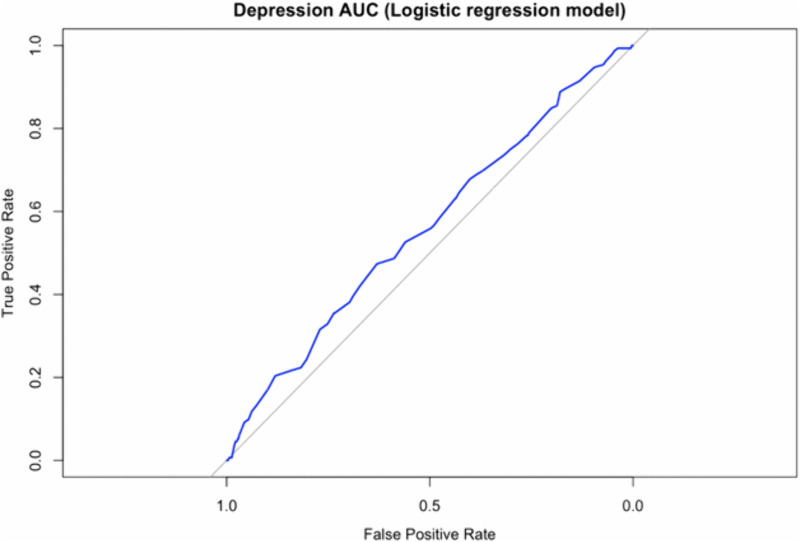
Receiver operating characteristic (ROC) curve for the logistic regression model predicting depression.

For depression, variables were selected based on p values < 0.05 in the logistic regression, resulting in geographical location (north and central area), current living situation (townships and villages), income level (overall), and living arrangement (“Who do you live with?”: husband, parents, and others) as significant predictors. These factors were included in the logistic regression model (AUC = 0.567). (Fig 4)

The model included the following significant predictors (p < 0.05): geographical location (north and central areas), current living situation (townships and villages), income level (overall), and living arrangement (husband, parents, and others). The area under the curve (AUC) was 0.567.

## 4. Discussion

This study is the first large-scale cross-sectional survey in Southwest China to integrate assessments of insomnia, anxiety, and depression among pregnant women. To better understand the findings, we employed a biopsychosocial model (BPSM). This model posits that mental health during pregnancy result ffrom the interplay of biological, psychological, and social factors. The model highlights the link between insomnia, anxiety, and depression: insomnia may exacerbate anxiety through biological pathways such as cortisol dysregulation, while anxiety may lead to depression through pathways such as psychological rumination and social isolation.

### 4.1 Insomnia among antenatal women

Our multivariate analysis revealed that the prevalence of insomnia symptoms among pregnant women was 28.4%. This figure is higher than that reported for Guangzhou (24.3%), a developed city [75], but lower than the prevalence in Lianyungang (59.8%), a moderately developed city [18].The adjusted odds ratios (aORs) indicated that the odds of insomnia were higher in the central and southern regions compared to the northern region, and lower in rural areas. Additionally, factors such as higher education levels, having more children, and living with someone other than the one's husband were associated with higher odds of insomnia [76]. After adjusting for potential confounders, no significant association was found between age, residence, income level, pregnancy status, employment status, gestational age, or expected mode of delivery and insomnia.

When comparing our findings with those from other regions, notable differences emerge based on sociodemographic contexts. In developing countries like India and Brazil [77], insomnia during pregnancy ranges from 33.5% to 54.4% [78], with higher rates attributed to factors such as poverty, malaria, partner violence, and limited mental health awareness, which worsen physiological vulnerability (e.g.,hormonal changes) and social stress (e.g., inadequate healthcare). In Malawi and Ethiopia [79], poor sleep quality is linked to these factors [80], with chronic inflammation and socioeconomic stress contributing to higher insomnia rates by affecting cortisol regulation. In contrast, in developed countries like the United States and the United Kingdom, insomnia in late pregnancy is around 42.4% [81], primarily associated with obstetric complications and urban environmental stressors such as noise pollution [82]. Studies show that employment or maternity leave reduces insomnia risk, similar to our findings, but higher education levels are associated with increased insomnia, possibly due to work stress. Developed countries offer better healthcare, mitigating biological risks (e.g., access to prenatal care), while developing countries face stronger social determinants of health that contribute to higher insomnia prevalence. Our finding of lower insomnia risk in rural areas may reflect reduced environmental noise, although this benefit may be offset by unmeasured factors such as infection status.

### 4.2 Anxiety among antenatal women

Our multivariate analysis revealed that the prevalence of anxiety symptoms among pregnant women was 4.6%, lower than the 17.4% reported in a meta-analysis in mainland China [83]. In developing countries such as Bangladesh, India, and Pakistan, pregnancy-related anxiety affects 20–30% of pregnant women, primarily due to insufficient social support, pregnancy-related stress, poor sleep quality, and medical conditions such as hyperthyroidism [84]. Research suggests that these patterns can be explained through biopsychosocial interactions, including biological factors (hormonal fluctuations), psychological factors (fear of childbirth), and social factors (economic disadvantage and domestic violence) [85]. Anxiety levels are often higher in rural areas because of geographic isolation and limited healthcare resources [86]. In developed countries such as Europe and the United States, pregnancy-related anxiety affects 10–18% of women [87], with associations to low education/social status, abuse history, and lack of partner support. Older age may reduce risk through maturity, which aligns with our findings [88]. Comprehensive mental health screening in developed countries reduces undiagnosed cases, while developing countries face social stigma and healthcare barriers, contributing to differences. Our lower prevalence may reflect underreporting, which is a limitation, though multivariate analysis strengthens causal inference compared to bivariate analysis [89].

### 4.3 Depression among antenatal women

Our multivariate analysis showed that the prevalence of depression symptoms among pregnant women was 8.44%, higher than the lifetime prevalence in the general population in China (3.4%) [90], but lower than the overall prevalence among pregnant women in mainland China (19.7%) [91]. Globally, antenatal depression places a substantial burden on low- and middle-income countries, including China, where the prevalence reaches 19.7% [91]. Research indicates that biological factors (e.g., malnutrition and infection), psychological stress, and lack of social support exacerbate depressive symptoms during pregnancy [92] [93]. In India and Brazil, the prevalence of antenatal depression exceeds 20% [94], with economic inequality contributing to the issue. In developed countries such as the United States and the United Kingdom, prevalence rates range between 10 and 15% [95], with similar risk factors but mitigated by available resources. For example, income can act as a protective factor through access to healthcare, consistent with our findings. The higher prevalence in developing countries is driven by systemic inequality, whereas our regional findings highlight comparatively lower risk in economically developed Chinese cities such as Shenzhen (7.3–10.9%) [96,97].

### 4.4 Clinical implications and potential interventions

The final structural equation model establishes a clear causal pathway linking insomnia, anxiety, and depression in this cohort of pregnant women. Insomnia emerged as the primary upstream factor (β = 0.741 to anxiety, p < 0.001), which in turn strongly predicts depression (β = 0.732, p < 0.001), with a smaller direct insomnia-to-depression path remaining (β = 0.138, p < 0.001). This chain-mediated structure provides practical clinical guidance. Routine antenatal care in Southwest China should prioritize systematic screening for insomnia, as it represents a modifiable entry point in the pathway. First-line treatment should include pregnancy-safe cognitive behavioral therapy for insomnia (CBT-I), delivered in group or digital formats, which meta-analyses show simultaneously reduces anxiety and depressive symptoms [98]. High-risk groups identified in our analyses, women in central and southern regions, urban residents, those with higher education, multiparous women, and women not living with their husbands, should receive targeted psychoeducation and enhanced social support. In particular, family-based interventions involving husbands and in-laws may strengthen protective social factors. At the policy level, extending mental health services during maternity leave and improving perinatal mental health infrastructure in less-developed regions may help address the geographic disparities observed in this study [30]. 

### 4.5 Interpretation of mental health category changes

Our analyses revealed variations in mental health categories driven by interconnected pathways. For example, SEM result indicates that insomnia (prevalence 28.4%) acts as an upstream factor, increasing the likelihood of subsequent anxiety (4.6%) and depression (8.4%), with partial mediation through anxiety (β = 0.732, p < 0.001). Sociodemographic covariates exacerbate these transitions. For instance, residence in the central region increased insomnia risk (OR = 1.818), potentially shifting women toward comorbid psychological states through increased stress exposure. These findings, supported by pairwise correlations, highlight dynamic interactions within the biopsychosocial framework and inform preventive strategies.

This study has several key strengths. First, the large sample from Southwest China enhances statistical power and representativeness. Second, simultaneous evaluation of insomnia, anxiety, and depression using validated measurement scales enabled the assessment of comorbidity patterns. Third, the use of multivariate logistic regression and structural equation modeling provided detailed insights into risk pathways and potential intervention targets across geographical, household, and socioeconomic domains.

However, several limitations should be noted. First, the cross-sectional design precludes causal inference. Second, reliance on self-reported questionnaires may introduce reporting bias. Third, the regional focus may limit generalizability to other populations. Fourth, the sample included a relatively high proportion of urban residents, potentially introducing sampling bias because urban women are more likely to access antenatal care services. Additionally, the relatively low explanatory power of the models suggests that other factors (e.g., biological markers or environmental stressors) may contribute to mental health outcomes. Finally, the predictive models may be sensitive to class imbalance and missing data, indicating that future studies could benefit from longitudinal designs or advanced machine-learning approaches.

## 5. Conclusion

This large-scale study in Southwest China identified significant rates of insomnia (28.4%), anxiety (4.6%), and depression (8.44%) among antenatal women. The key risk factors included marital status, spousal relationships, geographical location, and family dynamics, while family support appeared to function as a protective factor. These findings underscore the importance of targeted mental health screening and personalized interventions during pregnancy, particularly for women with identified demographic and socioeconomic risk factors, in order to improve maternal psychological well-being. 

## Supporting information

S1 Appendix Inclusivility global research. (DOCX)
