# Supplementary material for: Factors associated with insomnia, anxiety, and depression among antenatal women in China: A cross-sectional hospital-based study
Source: PLoS One. 2026 Mar 24;21(3):e0344846. doi: 10.1371/journal.pone.0344846 (PMC13012504; doi:10.1371/journal.pone.0344846)
Supplement: S1 Appendix — (DOCX) [file pone.0344846.s001.docx]

Inclusivity in global research

PLOS’ policy on inclusivity in global research aims to improve transparency in the reporting of research performed outside of researchers’ own country or community and ensures that PLOS publications reporting global research adhere to high standards for research ethics and authorship. Authors of relevant research articles may be asked to complete the questionnaire below, which outlines ethical, cultural, and scientific considerations specific to inclusivity in global research. This questionnaire may be requested when researchers have travelled to a different country to conduct research, if research uses samples collected in another country, research with Indigenous populations or their lands, or if research is on cultural artefacts. Researchers travelling to another country solely to use laboratory equipment will not normally be required to complete the questionnaire. However, the questionnaire can be requested at the journal’s discretion for any submission – if you have been requested to complete this questionnaire by the PLOS journal you submitted to, please do so.

Please complete the questionnaire below and include this as a Supporting Information file with your manuscript. Note that if your paper is accepted for publication, this checklist will be published with your article in the supporting information files. Please ensure that you reference the checklist in the main body of your manuscript. We suggest adding a subsection ‘Inclusivity in global research’ to your Methods section and adding the following sentence: “Additional information regarding the ethical, cultural, and scientific considerations specific to inclusivity in global research is included in the Supporting Information (SX Checklist)”

The questions have been designed to be applicable to a wide range of study types, and there are subsections for both human subjects research and non-human subjects research. If any of the questions are not relevant to your research please mark them as “N/A” as appropriate.

**Ethical considerations, permits and authorship**

*This section is applicable to all research types.*

Provide details as to who granted permissions and/or consent for the study to take place in the Methods section of your manuscript. This should include the names of **all** ethics boards, governmental organizations, community leaders or other bodies that provided approval for the study. If individuals provided approval refer to these people by their role or title but do not list their name(s).

Reported on page number: 8(Number 254-257)

If there were any deviations from the study protocol after approval was obtained please provide details of these changes in the Methods section of your manuscript.
Did this study involve local collaborators that are residents of the country where the research was conducted or members of the community studied? If you do not have any authors from said communities, please provide an explanation for this below.

Reported on page number: None

Everyone listed as an author should meet PLOS’ criteria for authorship and all individuals who meet these criteria should be included in the author byline, rather than the acknowledgements. For further information please see the journal’s Authorship Policy.

Yes, this study involved local collaborators who were residents of the country where the research was conducted and members of the community studied. These collaborators played an essential role in data collection, participant recruitment, and ensuring that the research was culturally relevant and ethically conducted. Their involvement helped ensure that the study accurately reflected the local context and community dynamics. All local collaborators who contributed significantly to the research, meeting PLOS' criteria for authorship, have been listed as authors in the manuscript rather than in the acknowledgements section, in accordance with the journal’s authorship policy.

**Human subjects research (e.g. health research, medical research, cross-cultural psychology)**

Did you obtain written informed consent from a representative of the local community or region before the research took place? How did you establish who speaks for the community? Details of written informed consent obtained from study participants should be reported separately in the Methods section of your manuscript.

Yes, written informed consent was obtained from a trusted representative of the local community before the research began. The process of identifying the appropriate representative involved consultations with local community leaders and key stakeholders, ensuring that the selected individual or group was recognized and supported by the community. This representative was entrusted with the authority to give consent on behalf of the community. They were involved throughout the study to ensure that the research aligned with the community's values, needs, and expectations. The process was conducted in a culturally sensitive manner, ensuring that the consent procedure respected local traditions and customs.

How did members of the local community provide input on the aims of the research investigation, its methodology, and its anticipated outcome(s)?

When engaging with the local community, how did you ensure that the informed consent documents and other materials could be understood by local stakeholders?

In engaging with the local community, we took several steps to ensure that the informed consent documents and other materials were easily understood by all stakeholders. First, we used plain language to avoid technical jargon and ensured that the terminology was culturally relevant and easily understood. Additionally, we provided visual aids where necessary to enhance comprehension, especially for abstract or complex concepts. Before data collection began, we conducted brief information sessions with community representatives to explain the research objectives, methodology, and expected outcomes in person. This allowed for immediate questions and clarifications, ensuring that all stakeholders were well-informed and fully understood the research. This approach helped make the informed consent process transparent, inclusive, and accessible, enabling participants to make informed decisions about their involvement.

Members of the local community provided input on the aims of the research, its methodology, and its anticipated outcomes through a series of consultation meetings, focus group discussions, and one-on-one interactions with community representatives. During these sessions, we used plain language to avoid technical jargon and provided visual aids where necessary to enhance understanding. Before data collection began, we ensured that community representatives had a clear understanding of the research objectives, methods, and expected outcomes, allowing for meaningful feedback. This collaborative approach allowed the community to raise concerns, offer suggestions for adjustments to the methodology, and align the research with their needs and values. This input directly influenced the final research design, ensuring it was culturally appropriate and contextually relevant.

Will the findings of the research be made available in an understandable format to stakeholders in the community where the study was conducted (e.g. via a presentation, summary report, copies of publications, etc.)? Please provide details of how this will be achieved.

Yes, the findings of the research will be made available to stakeholders in the community where the study was conducted in an understandable and accessible format. A summary report will be created that clearly highlights the key outcomes and their implications for the community. This report will be written in plain language to ensure clarity and avoid technical jargon. Additionally, visual aids, such as graphs and charts, will be included where appropriate to facilitate understanding, especially for complex findings.

**Non-human subjects research using specimens/ animals collected as part of the study, or those housed in archival collections. Examples include archaeology, paleontology, botany and zoology.**

Did the permission you obtained from a local authority to perform the study include an agreement on access to outputs and benefit sharing? This may include procedures to enable fair distribution of the benefits and resources arising from the research performed. Please include any details of Prior Informed Consent and Benefit Sharing Agreements obtained. These may be required by field-specific regulations, for example the Convention on Biological Diversity (CBD) and the associated Nagoya Protocol.

none

If the material used in your study was imported, please A) provide the year it was imported and B) indicate whether permits were obtained to import/export the materials used, C) provide details of any permits obtained. If this information is not available, please indicate this.

none

If you used archival specimens, please state how the material used in your study was acquired by the institute it is held in and provide details of any permits obtained for the original excavations/ sample collection. If this information is not available, please indicate this.

none

How was the potential cultural significance of the materials collected in your study to local communities considered in your research design? Were Indigenous peoples and/or local researchers and institutions involved with archaeological excavations / collection of specimens? If so, please provide a description of their involvement.

none

If your manuscript includes photographs of human remains please indicate whether authors obtained permission from descendants or affiliated cultural communities to do so.

none
